# Composite mantle cell and Burkitt lymphoma: a rare case report

**DOI:** 10.1097/j.pbj.0000000000000257

**Published:** 2024-06-20

**Authors:** José Guilherme Freitas, Ângelo Rodrigues, Susana Lisboa, José Mário Mariz

**Affiliations:** a Onco-Hematology Department, Portuguese Oncology Institute, Porto, Portugal; b Anatomic Pathology Department, Portuguese Oncology Institute, Porto, Portugal; c Genetic Department, Portuguese Oncology Institute, Porto, Portugal

Composite lymphoma (CL) is a rare entity defined by two or more morphologically distinct types of lymphoma (Hodgkin [HL] and non-Hodgkin lymphoma [NHL], B-cell lymphoma, T-cell lymphoma, or a mixture of B-cell and T-cell) within a single organ or tissue. The definition of CL is a matter of debate because it is difficult to prove simultaneous occurrence of two unrelated lymphomas and that CL is not just a clonal evolution such as histologic transformation, whose treatment approach is doubtful but are essentially targeted against the high-grade component.^1^ This case report, as far as we know, describes the first case of CL of Burkitt and mantle cell lymphoma.

Composite lymphoma is a rare entity defined by two or more morphologically distinct types of lymphoma (HL and NHL, B-cell lymphoma, T-cell lymphoma, or a mixture of B-cell and T-cell) within a single organ or tissue.^2^ Most cases of CL, the exact etiology of which is unknown,^1,3^ are two distinct types of B-cell lymphomas (usually a low-grade B-cell lymphoma), whose treatment approach is doubtful but are essentially targeted against the high-grade component.^1^ This case report, as far as we know, describes the first case of CL of Burkitt and mantle cell lymphoma.

A 68-year-old man presented with complaints of swelling in the right leg a month ago. The patient denied other symptoms, including B symptoms. Physical examination showed cervical and axillary adenopathies and a large inguinal mass associated with marked swelling of the right leg and scrotum. A computed tomography (CT) scan revealed supra and infradiaphragmatic lymph nodes, the largest lymph nodes involving the right iliac obturator with 14 cm. Initial blood work showed a Hb of 14.8 g/dL, WBC of 8.21 × 10^9^/L with a normal differential and a Plt of 237 × 10^9^/L, normal renal and hepatic function, and lactate dehydrogenase level of 216 U/L.

**Figure 1.**
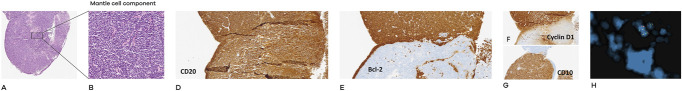
(A) (H&E 40X) biopsy sample from right inguinal mass showing two distinct cell populations: (B) (H&E 200X) on the upper half of the picture, there is a predominance of small cells, with centrocyte-like appearance, consistent with mantle cell lymphoma.

**Figure 2.**
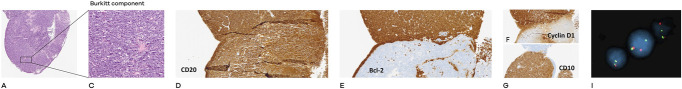
(C) (H&E 200X) on the lower half, the cell population is mostly comprised medium size cells with round nuclei containing multiple small nucleoli and apoptosis, compatible with Burkitt lymphoma. (D–G) Immunohistochemistry highlighting the main differences between the two components (D—CD20, E—Bcl-2, F—cyclin D1, G—CD10). FISH analysis showing IGH-CCND1 fusion signals (yellow) in the MCL component (H) and separate red and green signals in the Burkitt component with c-MYC rearrangement (I).

A biopsy of the right inguinal mass was performed, and pathologic examination revealed a composite lymphoid population (Fig. 1A). One population consisted of small cells (Fig. 1B) positive for CD20 (Fig. 1D), CD5 (focal), Bcl-2 (Fig. 1E), and cyclin D1 (Fig. 1F), and negative for CD3, CD10, Bcl-6, and CD23. Ki-67 was expressed in 20–30% of the cells, and fluorescent in situ hybridization (FISH) analysis was positive for rearrangement of CCND1/IGH in this population, resulting from the t(11; 14) (q13; q32) translocation, consistent with a diagnosis of mantle cell lymphoma (Fig. 1H). The other component was composed by intermediate/large cell population (Fig. 2C) positive for B-cell antigens CD20 (Fig. 1D), CD10 (Fig. 1G), and Bcl-6 and negative for CD3, CD5, CD23, cyclin D1 (Fig. 1F), and Bcl-2 (Fig. 1E). Ki-67 was expressed in >90% of the cells. *c-MYC* rearrangement was detected, supporting a diagnosis of Burkitt lymphoma (Fig. 2I). Bone marrow biopsy revealed an involvement of small CD20-positive lymphocyte population. Hence, we reached the final diagnosis of composite Burkitt and mantle cell lymphoma.

Before the initial treatment with chemotherapy R-CHOP protocol (rituximab, cyclophosphamide, vincristine, doxorubicin, and prednisolone), the patient was diagnosed with SARS-CoV-2 infection, without symptoms, and postponed the treatment one month.

After two cycles of R-CHOP protocol without clinical response, the treatment was suspended and a second line with R-HyperCVAD protocol (alternate cycles of a chemotherapy drug combination of rituximab, cyclophosphamide, doxorubicin, vincristine and prednisone, and methotrexate and cytarabine) was initiated. After the 3rd cycle, an atrial fibrillation with heart failure was diagnosed and the treatment was suspended. A bone marrow evaluation was performed and did not show any evidence of lymphoproliferative disorder, and a PET-CT did not show FDG-avid lesions. The patient is currently in clinical surveillance without clinical symptoms.

This case reports the first documented composite Burkitt and mantle cell lymphoma. The majority of components of CL were clonally related, suggesting a common origin.^4,5^ In fact, the definition of CL is a matter of debate because it is difficult to prove simultaneous occurrence of two unrelated lymphomas and that CL is not just a clonal evolution such as histologic transformation (eg. FL or CLL to DLBCL).^3,6^ In our case, aside the different morphologies in two components, the FISH analysis of the mantle cell component was negative for rearrangement of the *BCL6*, *MYC,* or *BCL2* genes and the Burkitt component was negative for the *BCL6*, *BCL2,* and *CCND1*/*IGH* rearrangements. This result supports the co-existence of two cytogenetically independent lymphomas.

Management of CL is extremely challenging, as there is no known standard of care treatment. In our case report, there was not any response with R-CHOP protocol, but the clinical and imaging improvement with addition of cytarabine and methotrexate highlights that HyperCVAD protocol appears to be an effective choice for CL of Burkitt and mantle cell lymphoma. However, long-term follow-up is necessary in our case to determine the outcome of this treatment.
